# The Effectiveness of Aromatherapy for Depressive Symptoms: A Systematic Review

**DOI:** 10.1155/2017/5869315

**Published:** 2017-01-04

**Authors:** Dalinda Isabel Sánchez-Vidaña, Shirley Pui-Ching Ngai, Wanjia He, Jason Ka-Wing Chow, Benson Wui-Man Lau, Hector Wing-Hong Tsang

**Affiliations:** Department of Rehabilitation Sciences, The Hong Kong Polytechnic University, Kowloon, Hong Kong

## Abstract

*Background*. Depression is one of the greatest health concerns affecting 350 million people globally. Aromatherapy is a popular CAM intervention chosen by people with depression. Due to the growing popularity of aromatherapy for alleviating depressive symptoms, in-depth evaluation of the evidence-based clinical efficacy of aromatherapy is urgently needed.* Purpose*. This systematic review aims to provide an analysis of the clinical evidence on the efficacy of aromatherapy for depressive symptoms on any type of patients.* Methods*. A systematic database search was carried out using predefined search terms in 5 databases: AMED, CINHAL, CCRCT, MEDLINE, and PsycINFO. Outcome measures included scales measuring depressive symptoms levels.* Results*. Twelve randomized controlled trials were included and two administration methods for the aromatherapy intervention including inhaled aromatherapy (5 studies) and massage aromatherapy (7 studies) were identified. Seven studies showed improvement in depressive symptoms.* Limitations*. The quality of half of the studies included is low, and the administration protocols among the studies varied considerably. Different assessment tools were also employed among the studies.* Conclusions*. Aromatherapy showed potential to be used as an effective therapeutic option for the relief of depressive symptoms in a wide variety of subjects. Particularly, aromatherapy massage showed to have more beneficial effects than inhalation aromatherapy.

## 1. Introduction

Depression is a life-threatening mood disorder manifested as a combination of cognitive and physical symptoms that leads to decreased interest in daily life activities [1] which imposes significant negative impact on people's quality of life and work performance due to disability, suffering, and high risk of perpetrating self-harm [2, 3].

Depression is reported as the largest health concern in the 21st century [4]. About 350 million people are currently suffering from depression [5]. Major depressive disorder has been projected to be the highest cause of years of life lived with disability by 2030 [6, 7]. The prevalence of depression has increased dramatically at a global level and one million people with depression commit suicide yearly [6, 8, 9]. In USA, an annual economic loss around USD 210 billion is associated with depression, which is one of the diseases with highest economic burden [10, 11]. Depressive symptoms include feelings of guilt, sadness, worthlessness and desperation, inability to experience pleasure, changes in appetite and sleep patterns, lack of energy, poor concentration and memory, motor retardation, fatigue, and recurrent suicidal and death ideation which are experienced for more than 2 weeks [1, 9, 12, 13].

Diagnosis of depressive symptoms is carried out using validated tools [14]. One of the oldest and most widely used diagnostic tools is the Hamilton Depression Rating Scale [15] which comprises a clinical-rated and a self-reported assessment. Another tool included the Beck Depression Inventory that uses a patient self-reporting tool of depressive symptoms [14]. These tools demonstrated high sensitivity and specificity and therefore are the best validated scales used for assessing the degree of severity of depressive symptoms [14, 16, 17].

Nowadays, the first-line treatment for major depressive disorder is antidepressants including monoamine oxidase inhibitors, tricyclic antidepressants, and serotonin-norepinephrine and selective serotonin reuptake inhibitors (SN/SSRIs) [13, 30, 31]. Despite the wide variety of antidepressants available in the market, a significant proportion of patients cannot reach full remission or experience side effects [13, 32, 33]. For instance, it has been reported that nearly 30% of the patients do not respond [8]. Side effects including nauseas, insomnia, agitation, weight gain, somnolence, sexual dysfunction, and cardiovascular adverse events have been reported [9, 31, 34]. Another downside of the use of antidepressants is the long treatment period needed to experience the beneficial antidepressant effect. Due to the ineffectiveness of the treatment in some patients or intolerability to the side effects, a large number of patients do not comply with the treatment and search for other therapeutic options [13, 31, 35]. Hence, increasing number of people with depressive symptoms explored other nonpharmacological interventions including psychotherapy and counseling, psychoeducation, exercise, problem solving therapy, guided self-help and behavioral activation treatments [36], or even complementary and alternative medicine (CAM) [3, 31].

CAM is defined as a broad set of healing resources, such as medical products and practices, for the prevention, diagnosis, and treatment of diseases that functions as a complement to the mainstream medicine system [37]. In USA, about 53.6% of the patients suffering from depression have reported to use CAM as an adjuvant therapy for the treatment of depression [31, 38]. One of the CAM options that patients with depressive symptoms choose is aromatherapy. Aromatherapy is defined as the therapeutic use of plant-derived concentrated essences which are extracted by distillation [39–41]. Aromatherapy is an inexpensive and noninvasive modality of CAM used to improve the psychological health and wellbeing [40, 42, 43]. Essential oils contain volatile organic compounds that exert a pharmacological effect by penetrating the body by oral, dermal (aromatherapy massage or topical application of aromatherapy) [44, 45], or olfactory administration (inhalation aromatherapy) [46–48]. The classification of essential oils is based on the botanical classification of the plant from which the essential oils are extracted [44]. The use of chemotypes is another classification of essential oils based on the subspecies of a plant with the same morphological characteristics that produces essential oils with different chemical profile, for example, type and quantity of chemical components [45]. The chemotype describes the main compound within certain essential oil [45]. Frequently, essential oils are used at different concentrations depending on the route of administration: (1) for aromatherapy massage, 1–5% essential oil is used, (2) for oral administration, 8–50% essential oil is used, and (3) concentrated essential oil is used in inhalation aromatherapy [48]. However, the dosage and dilution of essential oil chosen are not standardized in practice [48]. The most potent and effective administration method is oral administration in which the components of the essential oil reach the bloodstream [44]. Since essential oils are lipophilic, they can easily be carried to all organs in the body [44]. In inhalation aromatherapy, the inhaled air containing essential oils can not only reach the circulation system via the blood capillary network in the nose and the bronchi in the lungs but also stimulate brain areas directly via the olfactory epithelium [44, 48]. Essential oils trigger mechanisms in the brain via the olfactory system. The mechanism of action of essential oils administered by inhalation involves stimulation of the olfactory receptors cells in the nasal epithelium, about 25 million cells, connected to the olfactory bulb. After stimulation, the signal is transmitted to the limbic system and hypothalamus in the brain through the olfactory bulb and olfactory tract. Once the signals reach the olfactory cortex, release of neurotransmitters, for example, serotonin, takes place which results in the expected effect on emotions related to essential oil use [49–51].

Increasing popularity of aromatherapy has been reported in the UK as one of the most commonly used CAM therapies [52]. Due to the increasing popularity of aromatherapy this modality of CAM was chosen to carry out a systematic review on its effectiveness [53].

There was one published systematic review evaluating the effects of aromatherapy for people with depressive symptoms which included studies from 2000 to 2008 [2]. Since 2009 to date, 10 new RCT studies have been carried out to evaluate the effectiveness of aromatherapy on depressive symptoms thereby raising the need to update the discussion on the new findings taking into account all the evidence reported up to date on the topic. Therefore, this systematic review aims to provide an updated analysis of the evidence of the efficacy of aromatherapy for depressive symptoms.

## 2. Methods

### 2.1. Search Strategy

An extensive literature search was carried out in the following databases: Allied and Complementary Medicine Database (AMED), Cochrane Central Register of Controlled Trials (CCRCT), Cumulative Index to Nursing and Allied Health (CINAHL), MEDLINE, and PsycINFO. The predefined search strategy used to obtain the reference list of potential articles in the present study is shown in Table 1. Only studies in English were included and the search was carried out by 2 independent authors having a third author to consult when discrepancy occurred. The present study included randomized clinical trials involving adult subjects of both genders. There was no age restriction.

### 2.2. Inclusion and Exclusion Criteria for Study Selection and Outcome Measures

The studies included in the present review comprise RCTs with any kind of study design (e.g., double blind, single blind, and crossover study). No time restriction on the publishing year was considered for the study selection and studies that fulfill the inclusion and exclusion criteria up to date were included. Studies in which depressive symptoms were evaluated using any standardized assessment tool for depressive symptoms were included disregarding the type of clinical condition studied. Studies that assessed depressive symptoms by anxiety scales or any other assessments for depressive symptoms, for example, Profile of Mood States rating scale (POMS) or Hospital Anxiety and Depression Scale (HADS), were included. Eligible studies had to include the use of essential oils administered by inhalation or topical administration. Any study combining aromatherapy and massage was included regardless of the application method of the massage. There was no restriction in the duration of the treatment and number of sessions used. Systematic reviews and meta-analyses on aromatherapy and depression, mood disorders, or depressive symptoms were not included.

### 2.3. Selection of Relevant Studies

After the article search and removal of duplicates, the titles of the articles retrieved in the database search were screened. The abstracts of the preselected articles were screened to make a selection for further analysis. In case of doubt to include any study in the second screening, the full article was reviewed. Two independent authors carried out the search and selection of relevant studies for the present review. Disagreement was resolved by discussion.

### 2.4. Data Extraction

The data extracted included the reference, type of study, total number of subjects, number of subjects per treatment condition, brief description of the subjects, and the inclusion criteria. Regarding the intervention, information about the comparison group, type of aromatherapy, duration of the study, frequency of the treatment, outcome measures, and conclusion were extracted from the selected studies.

### 2.5. Quality Assessment

The quality of the studies included was assessed using the Jadad scale whose rating criteria take into account randomization, double blinding, withdrawals, and dropouts [54]. The scoring range in the Jadad scale goes from 0 to 5 in which a higher score represents higher quality of the study.

## 3. Results

### 3.1. Description of the Study Selection Scheme

**Figure 1:**
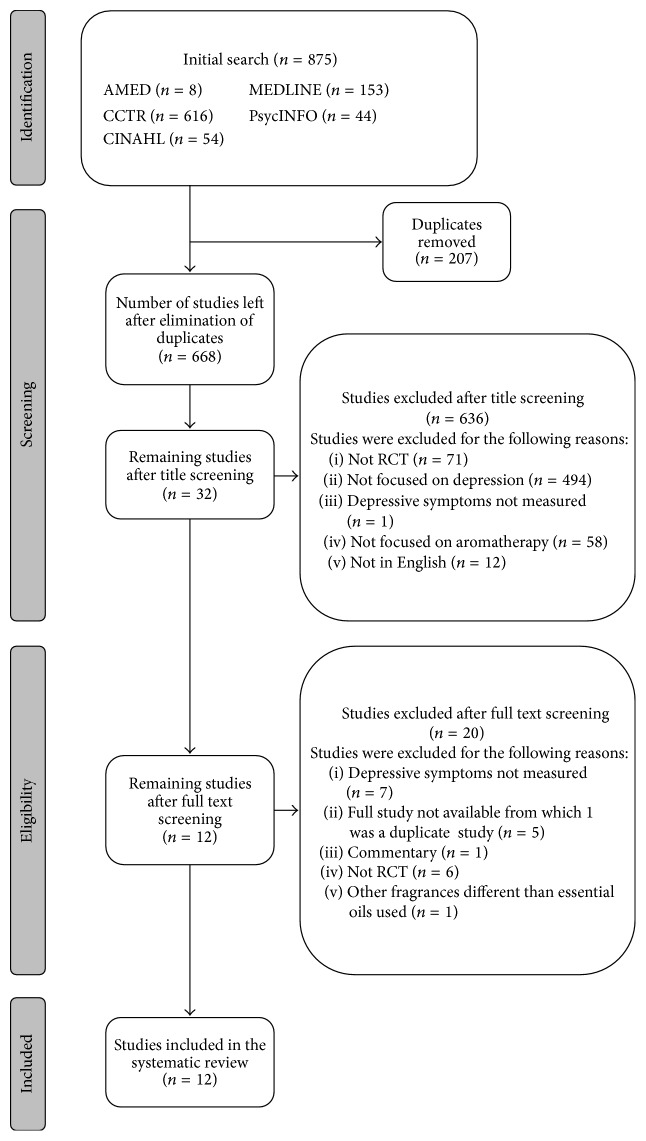
Study selection flowchart.

The combined database search was carried out from inception to May 2016 and resulted in 875 studies identified using the predefined search terms (Figure 1). After removal of duplicates (*n* = 207), the title of 668 studies was screened. Most of the studies excluded were not concerned with depression and/or aromatherapy (*n* = 552). In addition, 84 studies were excluded because they were not RCTs, they were not in English, and/or no depressive symptoms were measured. After title screening, 32 studies remained for further full text screening. A total number of 20 studies were excluded at this stage, 7 studies did not assess depressive symptoms, 1 study was a commentary, 6 were not RCTs, 1 study assessed colognes which are not essential oils, and 5 studies could not be accessed. From the 5 studies that could not be accessed, 1 study was not detected as duplicate before since it appeared with a short title in the database search; therefore, only 4 studies could not be accessed. The abstract of 2 of those studies was available while no abstract was available of the other 2 studies. The authors of those 4 studies were contacted via email requesting them the full studies. We could not get access to four studies whose title suggested that the studies could be included in the systematic review. However, the studies were nor provided by the author due to the following reasons: the study was still unpublished, the authors did not reply, or the author could not be reached. Therefore, those studies were excluded. A total number of 12 RCTs [18–29] that met the inclusion and exclusion criteria were selected.

### 3.2. Description of the Subjects

Detailed information on the subjects is stated in Table 2. The total number of subjects from all the studies was 1226 from which 984 were female (80.3%) and 224 (18.3%) were male participants. The study conducted by Lemon [23] did not specify the number of female and male subjects in the control group; therefore the gender of 18 subjects (1.4%) was not taken into account in the above mentioned percentages. The mean subject age included in the studies was 47 with mean average age range of 21–73. The participants included in the studies selected are people with cancer (*n* = 682), pregnant women (*n* = 333), women in menopause phase (*n* = 90), patients diagnosed with depression and/or anxiety (*n* = 32), postpartum women (*n* = 28), women whose children were diagnosed with attention deficit/hyperactivity disorder (*n* = 25), healthy female volunteers (*n* = 20), and patients diagnosed with idiopathic environmental intolerance (*n* = 16).

### 3.3. Intervention

#### 3.3.1. Control Group

The comparison groups used in the studies included no intervention group (*n* = 6) [20, 22, 24, 26, 28, 29], vehicle group (*n* = 4, received vehicle such as carrier oil or water) [18, 19, 21, 23], and active control group (*n* = 2; usual supportive care and cognitive behavior therapy, well known treatments with positive effect on the outcome measures) [25, 27].

#### 3.3.2. Intervention Group

Two administration methods for aromatherapy identified in the studies selected include aromatherapy via inhalation (inhalation aromatherapy, *n* = 5) [18–22] and aromatherapy with massage (aromatherapy massage, *n* = 7 plus 1 study from Conrad and Adams, 2012, which also used inhalation aromatherapy) [19, 23–29]. No RCT study included involved aromatherapy administered orally. Details of the intervention adopted in the 12 included studies were summarized in Table 3.

In the study carried out by Sehhatie et al. [22], a combination of nonpharmacological interventions for pain relief in labor, including aromatherapy, was used in the intervention group. The contribution of aromatherapy in the combined intervention cannot be discriminated in this study. Therefore, caution should be taken when discussing the results of this study.

### 3.4. Selection of Essential Oils

Essential oils were mainly used pure, diluted, or in a mixture of 2 or more essential oils at a particular ratio. The selection of the essential oils used was determined by the aromatherapist, the effect on physical and physiological states, subject's preference, or safety for use during pregnancy while other studies did not mention in the methodology section the rationale behind the essential oils chosen nor specify the type of essential oils used. The most commonly used essential oils were lavender in 8 studies [18–20, 22–24, 28, 29].

#### 3.4.1. Inhalation Aromatherapy

The essential oils that were more commonly used in the inhalation aromatherapy studies were lavender and bergamot either as a single essential oil or in a mixture with other essential oils [18–20, 22–24, 28, 29]. One mixture of fractionated essential oils was used, but the type of essential oils contained in the mixture was not specified [18]. In addition, the purity of the fractionated mixture was unknown. Other essential oils utilized were petitgrain [20] and Yuzu [21] essential oil alone while cedarwood [18] and rose otto [19] were used in combination with a mixture of lavender and bergamot, and lavender, respectively.

#### 3.4.2. Aromatherapy Massage

A set of 20 different essential oil options were used in two of the studies from which the therapists chose the most suitable essential oil for each one of the subjects [25, 27]. However, the type of essential oils used provided to the participants was not specified in those studies. On the other hand, in the study conducted by Lemon [23], the essential oils used were selected from a list of 9 options and the author specified the type of essential oil used in each subject of the intervention group. The essential oils used in the other studies comprise lavender, a mixture of 2–4 different essential oils, and rose otto combined with lavender.

### 3.5. Administration Protocol

#### 3.5.1. Inhalation Aromatherapy

The method of administration of inhalation aromatherapy also differed among the studies [18–22]. The main differences in the administration methods rely on the distance between the aroma source and the subject's nose. In one study, cotton impregnated with essential oil placed in a diffuser was set in the nostrils of the subjects [21]. In the other two studies the source of aroma was placed approximately 30 cm away from the nose of the subjects [18, 20]. In other two studies, the essential oil was applied to a bib or to a cloth that were worn by the participants [18, 22]. The volume of essential oil used in the inhalation aromatherapy studies varies from 10 *μ*L to 1 mL or 3, 5, or 8 drops. The exposure time to the aroma ranged from 5 to 20 minutes, with the number of sessions from 1 to 56 sessions [18–21] (Table 3). In one study, the total duration of the exposure to the aroma was not specified since the intervention was carried out during the active phase of the labor process that pregnant women underwent [22]. Furthermore, the frequency of the treatment in the 5 studies differs greatly. For example, the frequency of treatment in the studies varies from once [20, 22], twice [21], and twice a week [19] to daily [18]. The duration of the treatment in the inhalation aromatherapy studies allowed the evaluation of acute and long term effect due to the duration of the treatment from 1-2 days to 4–8 weeks, respectively.

#### 3.5.2. Aromatherapy Massage

The types of massage are performed with standardized protocols [24–27] in which 3 of the studies [25–27] did not describe the areas of the body for the delivery of the massage while another study specified the target body areas to deliver the massage such as back massage [24]. Other massage target areas were also described in three studies [19, 28, 29]. Taavoni et al. [28] focused the massage on the abdomen, thighs, and arms while Wu et al. [29] focused on the neck, shoulders, arms, back, and legs with effleurage, friction, petrissage, and vibration at a moderate pressure. In the study of Conrad and Adams [19], the essential oil mixture was applied on both hands (hand aromatherapy massage) using the well m'technique which involves gently stroking movements applied in a set sequence with structured strokes, sequence, number, and pressure [45]. The duration of the studies was 4, 8, 10, and 12 weeks and 2 years. Weekly sessions were carried out in most of the studies [19, 24, 25, 27–29]; only in one study the frequency was once every 2 weeks [26]. The number of sessions per week also varied from once or twice weekly to once every 2 weeks. The duration of the treatments was 15, 30 min, and 40 min to 1 h and the total number of sessions varied from 4 and 6 to 8 sessions.

### 3.6. Outcome Measures

A summary of the outcome measures is shown in Table 4. The most frequently used instruments were HADS and POMS [18, 20, 21, 24, 26, 27] followed by EPDS which was used in 2 studies [19, 22]. Other assessment tools include the MADRS [23], MRD [28], BDI [29], and CES-D [25].

### 3.7. Efficacy of Aromatherapy

#### 3.7.1. Inhalation Aromatherapy

Two out of 5 studies evaluating the effect of inhalation aromatherapy reported beneficial effects to improve depressive symptoms in the subjects [19, 21]. The subjects in the study of Conrad and Adams [19] were postpartum women exposed to two different aromatherapy interventions, inhalation aromatherapy and topic application of aromatherapy, for 8 sessions. At baseline, control and treatment groups showed similar levels of depressive symptoms (EPDS: *p* = 0.8). At the end of the study, the treatment group with aromatherapy showed a significant reduction of depressive symptoms (EPDS: *p* = 0.01), but the improvement was lesser than using m'technique. The study conducted by Matsumoto et al. [21] showed improvements in negative emotional stress after 2 sessions of 10 min on healthy volunteers. The TMD score (*p* < 0.001) and the depression-dejection (*p* = 0.041), tension-anxiety (*p* < 0.018), anger-hostility (*p* = 0.002), and confusion (*p* = 0.019) subscores decreased significantly after inhalation of yuzu, respectively.

The other 3 studies in which no beneficial effect of inhalation aromatherapy was observed included Graham et al. [18], Igarashi [20], and Sehhatie et al. [22]. According to the study conducted by Graham et al. [18], the percentage of individuals with cancer showed an increase in the outcome measurement when compared to the initial baseline, from 18 to 22% using HADS. Graham et al. [18] concluded that inhalation aromatherapy did not reduce the levels of depressive symptoms in people with cancer undergoing radiotherapy and receiving inhalation aromatherapy.

Two studies evaluated the acute effect of inhalation aromatherapy on pregnant women, but no statistically significant difference (*p* value not given) was observed [20, 22]. In the study by Igarashi [20], inhalation aromatherapy showed no decrease in the depression-dejection mood state in the intergroup comparison in the POMS scale. The acute effect of inhalation aromatherapy assessed in the study carried out by Igarashi [20] showed an improvement in profile of mood states among the subjects. However, no difference was found when comparing the scores of the inhalation aromatherapy group and the control group. On the other hand, tension-anxiety and anger-hostility mood states showed a significant decrease (*p* < 0.05; *p* < 0.05, resp.) in the aromatherapy group [20]. Aromatherapy showed to be effective in POMPS in intragroup comparison, but no difference was observed when comparing the groups in pregnant women.

Sehhartie et al. [22] found no statistically significant difference (*p* = 0.610) in the reduction of the degree of postpartum depression between the control and the intervention group. They concluded that nonpharmacological interventions, including inhalation aromatherapy, for pain relief in labor did not reduce the degree of postpartum depression.

#### 3.7.2. Aromatherapy Massage

Five out of 8 studies showed positive effects of aromatherapy when the intervention was carried out in combination with massage [23, 26–29]. In the study of Lemon [23], the assessment of depressive symptoms using MDRS showed slight improvement in the control group which was expected and attributed to the effect of massage alone. On the other hand, statistically significant difference was observed in the treatment group when comparing the baseline and end point scores. Lemon [23] concluded that aromatherapy massage had beneficial effect on subjects who showed mild or higher degree of depressive symptoms. Araki et al. [26] observed a statistically significant difference (*p* < 0.001) in the depression subscale of the POMS when comparing the baseline and end point scores. Although aromatherapy did not show to have any beneficial effect for the management of idiopathic environmental intolerance, the data reported showed that aromatherapy massage improved all the subscales of the POMS including depression. Conrad and Adams [19] who evaluated the effect of the application of aromatherapy on both hands in addition to the effect of inhalation aromatherapy showed a significant improvement (EPDS, *p* = 0.025) in depressive symptoms which was superior to the improvement observed when using inhalation aromatherapy. At the end of the study, a significant difference (EPDS, *p* = 0.003) was observed between the control and treatment group. Inhalation aromatherapy showed an improvement in the reduction of depressive symptoms but only when the data was presented as a combination of aromatherapy intervention including inhalation aromatherapy and aromatherapy massage. Therefore, caution should be taken when discussing these results. Another study that showed beneficial effects of aromatherapy massage was the study of Serfaty et al. [27]. The depression-dejection and tension-anxiety scores were similar in both interventions using the POMS-TMS. Aromatherapy massage showed to be advantageous in terms of general feeling in the last week and the cognitive behavior therapy seems to be more effective and the effect is sustained for a longer period according to the POMS-TMS and subscales scores. Serfaty et al. [27] concluded that both interventions showed the improvements and the effects of cognitive behavior therapy on depression showed to be sustained over time. Taavoni et al. [28] measured a series of psychological symptoms including depressive mood using the MRS. A statistically significant difference (*p* < 0.001) was found among the three groups evaluated. No difference was found between the pre- and posttest in the control group (Table 4). However, both massage and aromatherapy massage showed an improvement in the outcome measures. The conclusion of this study was that massage aromatherapy was more effective than massage to reduce the psychological scores. Finally, the study conducted by Wu et al. [29] showed a statistically significant difference (*p* = 0.04) in the treatment group when comparing the pre- and posttest depression assessment scores using the BDI while no significant difference (no *p* value given) was observed in the control group. Wu et al. [29] concluded that aromatherapy massage improves depressive symptoms.

The studies on aromatherapy massage that showed no beneficial effect of the intervention include Soden et al. [24] and Wilkinson et al. [25]. Soden et al. [24] did not find any significant difference in HADS among the control and aromatherapy massage groups. Similarly, Wilkinson et al. [25] reported no statistically significant difference in the levels of depression in people with cancer receiving aromatherapy massage combined with the usual supportive care intervention when compared with the usual supportive care intervention alone. Improvement in clinical depression was observed in 63% of the patients, but no difference in improvement was observed when comparing the two treatment groups. No significant difference in the self-reported depression was observed between the two groups. Wilkinson et al. [55] concluded that individuals with cancer receiving aromatherapy massage did not experience the reduction in the level of depressive symptoms.

### 3.8. Quality Assessment

Quality assessment of the included studies was graded by Jadad scale (Table 5). Jadad scale is a scoring system that assesses the quality of methodology of randomized controlled trials (RCTs). Jadad scale ranges from 0 to 5, with 5 comprising description of randomization (2 points), double blinding (2 points), and withdrawals and it is a frequently used tool for assessing the risk of bias in the studies in which low scores are associated with high effect estimates [54, 56]. Out of 12 studies, one study was rated 5 [24], two studies were rated 4 [18, 22], three studies were rated 3 [20, 25, 27], three studies were rated 2 [19, 26, 28], and three studies were rated 1 [21, 23, 29]. All the studies included were RCTs. Among 12 articles, 4 studies did not describe the method of randomization [19, 21, 23, 29]. In 6 studies, no blinding was used [19, 20, 23, 26, 28, 29]. Single blinding was used in 2 studies [21, 27]. Three studies were double blind studies [18, 22, 24, 25] and the description and appropriate double blinding procedure was provided in 2 studies [18, 24]. Proper description of dropouts and withdrawals was provided in 6 studies [19, 20, 22, 24, 25, 27].

## 4. Discussion

The objective of this systemic review was to analyze the clinical evidences on the efficacy of aromatherapy for depressive symptoms and to update the previously published systematic review by Yim et al. [2].

### 4.1. Comparison with the Previous Systematic Review

The objective of this systemic review was to analyze the clinical evidences on the efficacy of aromatherapy for depressive symptoms. Regarding the comparison between the previously published systematic review on the effectiveness of aromatherapy on patients with depressive symptoms and the present systematic review, an updated detailed analysis of the evidence on the topic is provided in the present analysis. In the limitations of the systematic review, the authors mentioned that the sample size of the studies selected was small and they highlighted the only 2 RCTs were included. From the date of publication of the systematic review from Yim et al. in 2009 [2] to the date that the present systematic review was carried out, a higher number of RCTs have been published and some of the limitations described by Yim et al. [2] were addressed in the present systematic review as discussed below. The difference in the number of RCTs included in both systematic reviews suggests that in recent years the need for high quality clinical evaluation of the effectiveness of aromatherapy has been of increasing interest.

Although the search terms used in both systematic reviews were similar, our main focus was on RCTs. Two studies that fulfilled the inclusion and exclusion criteria established in the present systematic review were also included in the previous systematic review carried out by Yim et al. [2]. Those 2 studies selected in both systematic reviews are Soden et al. [24] and Wilkinson et al. [25]. One limitation reported by Yim et al. [2] was the small sample size used in the studies included. However, the sample size of the studies included in the present systematic review was larger.

### 4.2. Effectiveness of Aromatherapy to Relieve Depressive Symptoms

The systematic review included 12 studies with a diverse type of subjects such as pregnant women, postpartum women, women in menopause phase, women with children diagnosed with attention deficit hyperactivity disorder (ADHD), healthy female volunteers, patients diagnosed with cancer, depression/anxiety, and idiopathic environmental intolerance. Discrepancy on the effectiveness of aromatherapy in both inhalation aromatherapy and aromatherapy massage was found. The mixed results could be related to the differences in the administration protocol or the diverse type of subjects included in the RCTs. In addition, caution should be taken when aromatherapy is administered by inhalation since proper olfactory function should be confirmed before starting the trial and the degree of the beneficial effect of aromatherapy when combined with massage should be properly studied to evaluate whether the added effect of aromatherapy is large enough to surpass the effect of massage alone.

Another important aspect to take into account to analyze the effectiveness of aromatherapy is the chemical nature of the different essential oils used in the studies. The chemical composition and mechanism of action of the essential oils used have shown beneficial effects on mood parameters such as anxiety, depression, and sedation which support their use in the clinical studies included [42, 57–59]. For instance, lavender [57], bergamot [58], and sandalwood [42] have shown to improve depressive symptoms while yuzu alleviates negative emotional stress [59]. The rest of the essential oils used contain chemicals such as limonene, linalool, and linalyl acetate that have been widely studied and have showed anxiolytic and sedative properties. On the other hand, 2 studies [25, 27] used a set of 20 essential oils but the type of essential oils used was not stated. Therefore, it is not possible to make an analysis on the nature of the essential oils used in those studies. Most of the studies used single, mixed, or diluted essential oils. Also, all the essential oils used, except the ones not specified in 2 studies, have shown to have anxiolytic, antidepressant, and sedative properties as stated above. Due to the differences in administration method, duration of the treatment session, frequency of the treatment, total number of sessions, and forms of essential oils (i.e., single, mixed, or diluted form), it is complicated to make a comparison of the treatment efficacy across different studies only taking into account the type of essential oil.

#### 4.2.1. Inhalation Aromatherapy

In the present systematic review, 5 out of 12 studies used inhalation therapy as a modality of aromatherapy. However, only the studies carried out by Matsumoto et al. [21] and Conrad and Adams [19] found beneficial effects of the essential oil yuzu and a mixture of rose otto and lavender, respectively, to relieve negative emotional stress and depressive symptoms.

Inhalation aromatherapy given to people with cancer showed no effect although the number of sessions used was high (56 sessions). The lack of efficacy could be due to the quality of the essential oils used since Graham et al. [18] reported that one intervention group was administered with unknown purity essential oils and the other group was administered with a mixture of 3 pure essential oils at different concentration ratio.

Two studies carried out on pregnant women were also included. The results from the study carried out by Igarashi [20] showed acute beneficial effect of inhalation aromatherapy to decrease depressive symptoms in pregnant women. However, the difference observed was the result of within group comparison. Furthermore, the duration of the treatment and frequency of the aromatherapy intervention in pregnant women were too short to allow a full evaluation of the effectiveness of the intervention. Another study carried out on pregnant women did not show any significant benefit from inhalation aromatherapy intervention [22]. Both studies on pregnant women only used one session of inhalation aromatherapy of 5 min in one study [20] and unknown exposure time in the other study [22]. Therefore, the lack of effectiveness reported could be associated with the short intervention exposure time in the treatment groups.

Another population in the present systematic review was postpartum women including a group receiving inhalation aromatherapy and another group receiving aromatherapy massage applied to both hands [19]. This study showed improvement in depressive symptoms of both interventions. However, the authors combined the data of both interventions to make the statistical analysis and concluded that both aromatherapy interventions together were effective. The data presented only supported the effectiveness of aromatherapy massage but not that of inhalation aromatherapy. However, no group receiving only hand massage without aromatherapy was included to assess the contribution of the hand massage alone on the effectiveness of the intervention. Hence, it is not possible to conclude that the effect was attributed to aromatherapy massage.

In the inhalation aromatherapy intervention on healthy volunteers, beneficial effect was observed. When comparing the protocols of studies with positive findings with the protocols of the studies that did not show positive effect, the lack of effectiveness may be due to distance between nostrils and aroma. In the study from Matsumoto et al. [21], which shows positive effect of aromatherapy, the intervention was administered using a diffuser placed in the subject nostrils. The proximity of the source of aroma to the nasal mucous may have enhanced the interaction between the volatile compounds of the essential oil and the olfactory receptors. In addition, Matsumoto et al. [21] carried out 2 sessions of 10 minutes prior to the RCT study to make sure that the olfactory ability of the subjects had normal olfaction. The presence of a pretest and short distance between nostrils and aroma might be the reason behind the difference of efficacy observed in different studies involving inhalation aromatherapy. Both cotton impregnation and diffuser were effective methods to bring beneficial effect [49, 60, 61].

#### 4.2.2. Aromatherapy Massage

Aromatherapy massage is another modality employed in 8 out of the 12 studies selected in which 5 studies showed positive effect of the intervention. Aromatherapy massage is a combination of aromatherapy and massage that offers the health benefits of both therapies and is commonly used by healthy individuals particularly for stress management [29]. Massage has been reported to be a popular therapy for the management of depression in which about 2.1% of the patients with severe depression undergo massage therapy for the relief of depressive symptoms [62] and this therapy has been used in palliative care settings and individuals with cancer [29].

In aromatherapy massage, difficulties to separate the effects due to aromatherapy from the health benefits due to massage bring concerns. Both therapies alone have shown to be effective in the alleviation of psychological symptoms. It is clear that each therapy has a complex multitarget approach which leads to the effect observed. The aromatherapy massage studies included in the systematic review comprised different massage techniques and some of the important information is missing. For instance, the area of the body part to massage may not be reported [25]. Furthermore, the catalogue of massage techniques is diverse and the decision on the type of massage to be used may be elusive. Some examples of massage techniques include (1) effleurage, (2) kneading, (3) petrissage, (4) friction, (5) tapotement, and (6) vibrations and shaking [63]. The type of massage used has been linked to different physiological effect, for example, increase of the blood flow, enhancement of venous return flow, increased cardiac stroke volume, and even production of shirt-lived analgesia [63]. Therefore, the details of the massage technique used are very important to analyze the evidence of the aromatherapy massage studies. On the other hand, not much information about the massage technique was provided in these studies thereby limiting a deeper analysis of these results.

The study that showed effectiveness of the intervention [27] involved twice the number of sessions than the massage therapy protocol of the studies with no effect of the intervention [24, 25]. Based on the results reported on people with cancer, aromatherapy massage is effective compared to the active control when the number of sessions is at least 8 and administered weekly. In addition, studies carried out on the effect of massage therapy in the relief of symptoms experienced by individuals with cancer showed a positive effect of massage alone which contributed to the alleviation of the symptoms [32]. The lack of efficacy observed in the aromatherapy massage studies included in the systematic review might be due to the effect of massage itself without added beneficial effect of aromatherapy when used in combination with aromatherapy [32, 64–66]. Furthermore, caution should be taken regarding the interpretation of massage therapies since the positive effects on symptom relief in people with cancer are not compelling [67]. On the other hand, Fellowes et al. [68] reported a positive but limited effect of aromatherapy massage for alleviation of symptoms in people with cancer which is a clear example of the careful interpretation of the results that has to be done and the need of more studies to overcome the mixed evidence involving aromatherapy massage. Furthermore, Serfaty et al. [27] reported positive results in the aromatherapy massage group. However, it should be noted that aromatherapy massage was not given alone but in combination with cognitive behavior therapy. As direct effect of aromatherapy massage alone was not assessed in the study of Serfaty et al. [27], the evidence suggests that aromatherapy massage is effective when given in combination with cognitive behavior therapy.

The other two studies involving women in the menopause phase [28] and women with children diagnosed with ADHD [29] showed positive results in aromatherapy massage. Both studies used similar protocols in which the intervention was administered in a total number of 8 sessions twice a week for 30–40 minutes. Other two studies with a different subject population in which the effect of aromatherapy massage was evaluated used lower frequency of treatment (once a week) and lesser number of sessions (4) and no effect of the intervention was observed [24, 25]. When comparing the protocol settings with the rest of the aromatherapy massage studies carried out on different subject populations, it is noted that a higher number of sessions (6–8 sessions for 40 min to 1 h per session) were used in the studies in which beneficial effect of aromatherapy massage was observed [23, 27]. Only in one study [26] with low frequency of treatment (every two weeks) and 4 sessions of 1 hour per session, beneficial effects of aromatherapy massage were observed. In summary, the data presented in aromatherapy massage suggest that at least more than 4 sessions weekly or twice a week for at least 30 minutes would provide good administration settings to increase the likelihood of observing positive results in aromatherapy massage. However, it is also important to take into account other factors such as the comparison group, massage technique, and essential oils used to draw a conclusion on the effectiveness of aromatherapy massage.

In other two studies on aromatherapy massage, the subject population corresponded to patients diagnosed with depression and/or anxiety [23] and patients with idiopathic environmental intolerance [26]. Both studies showed positive results in the relief of depressive symptoms and the comparison groups were massage without aromatherapy and no intervention with 4–6 sessions of aromatherapy massage for 40 minutes to 1 hour. In the study of Lemon [23] the improvement in the depression level in the control group was attributed to the effect of massage alone. However, the aromatherapy massage group also showed positive effect on the relief of depressive symptoms. In the study carried out by Araki et al. [26], significant beneficial effect was observed in the aromatherapy massage intervention.

## 5. Limitations

Six out of the 12 studies included in the systematic review scored low (score of 1 or 2) in the Jadad scale. Therefore, the poor blinding in those studies could have contributed to the perceived effect of the treatment and have a positive impact on the effectiveness of the treatment. In addition, the administration protocol varied considerable among the studies. Particularly, in aromatherapy massage, the massage technique used is not fully described in some of the studies while in other studies it is briefly described. In order to analyze the contribution of the massage alone in the aromatherapy massage intervention, understanding of the benefits of the massage technique applied is crucial. However, in some studies the massage protocol is not described or no massage comparison group was used to make the discrimination of the effect between massage alone and aromatherapy massage. Also, different assessment tools were used across the studies; therefore, comparison of the evidence among the studies is difficult. Finally, differences regarding changes or developments in the field of aromatherapy for the treatment of depressive symptoms when comparing both systematic reviews are difficult to highlight due to the diversity of the studies included in terms of subjects and intervention protocols.

## 6. Clinical Recommendation

When using inhalation aromatherapy, inclusion of a pretest is important to ensure that subjects have adequate olfactory function before the commencement of the treatment. Furthermore, a longer exposure time and higher number of sessions should be considered in the inhalation aromatherapy treatment since positive results were observed when a higher number of sessions and longer exposure times were used. Based on the protocols presented from the included studies, at least 8 sessions in the treatment are needed to assess the effectiveness of aromatherapy massage and beneficial effects to relieve depressive symptoms. In addition, it is suggested to apply aromatherapy massage treatment once or twice per week.

## 7. Conclusions

When Yim et al. [2] concluded that there was no sufficient evidence suggesting that aromatherapy could be used as complementary and alternative medicine for depression, the analysis of the evidence up to date presented in our systematic review showed otherwise. The present systematic review offers an overview of the most current evidence. Particularly, aromatherapy massage showed to be more efficacious than inhalation aromatherapy to alleviate depressive symptoms. However, inhalation aromatherapy also showed to be effective, but further studies will be needed to have more conclusive evidence on this aromatherapy modality. In the overall analysis carried out, aromatherapy showed potential to be used as an effective therapeutic option for the relief of depressive symptoms in a wide variety of subjects.

## Figures and Tables

**Table 1:** Search terms and database search strategy.

ID	Disease search terms
1	Depress^*∗*^
2	Major depress^*∗*^
3	Mood disorder
4	Depressive disorder
5	1 OR 2 OR 3 OR 4

ID	RCT search terms

6	Controlled clinical trial^*∗*^
7	Random^*∗*^ controlled trial^*∗*^
8	6 OR 7

ID	Aromatherapy search terms

9	Aroma
10	Aromatherapy
11	Aromatic therapy
12	Essential oil^*∗*^
13	Fragrance
14	Fragrant oil^*∗*^
15	Scent
16	Massage therapy
17	Medical massage
18	Massage
19	9 OR 10 OR 11 OR 12 OR 13 OR 14 OR 15 OR 16 OR 17 OR 18
20	5 AND 8 AND 19

^*∗*^Truncation symbol for database search.

**Table 2:** Characteristics of the participants included in the selected studies.

Ref.	Type of study	Subjects					
		Total number of subjects	Mean subject age (range)	Gender (*n*)	Type of subject	Diagnostic systems/inclusion criteria	Baseline score for depressive symptoms
*Inhalation aromatherapy*							

[18]	Placebo-controlled randomized double blind RCT	313	65 (33–90)	Female (150) Male (163)	Individuals with cancer receiving radiotherapy treatment	Patients prescribed with 8 or more fractions of radiotherapy	Baseline depression status: odds ratio of 29 using HADS.

[19]^*∗*^	Randomized observational pilot study with repeated measures	28	32 (25–43)	Female (28)	Postpartum women	0–18-month postpartum women with scores of 10 or higher on either the Edinburgh Postnatal Depression Scale or the Generalized Anxiety Disorder Scale	The baseline score using the Edinburgh Postnatal Depression Scale for the control group was 15.9 and 16.1 for the intervention group.

[20]	Prospective RCT	13	27.3 for the control group (NA) 29.3 for the treatment group (NA)	Female (13)	Pregnant women	28-week-pregnant women, singleton pregnancy	Depression-dejection scale baseline score using POMS was 2.7 in the control group and 1.6 in the treatment group.

[21]	Randomized controlled crossover study	20	20.5	Female (20)	College students	Healthy volunteers	The depression-dejection scale baseline score using POMS was not provided, but the change difference between pre- and posttreatment was reported. The change in depression-dejection score was lower than −1 in the treatment group and statistically significant when compared to the change in the control group.

[22]	Controlled double-blinded RCT	320	20–30, average age NA	Female (320)	Pregnant women	Women between 18–35 years, with a pregnancy age between 38 and 42 weeks, a score of 12 or less in the Edinburgh test	Depression grade baseline in the Edinburgh test was 6.3 in the control group and 6.1 in the intervention group.

*Aromatherapy massage*							

[23]	RCT	32	32.9 (23–53) in the treatment group	Female (10 in the treatment group) Male (4 in the treatment group) No information provided on the number of female and male subjects in the control group	Patients with depression and/or anxiety	Patients scoring more than 7 in the Montgomery-Asberg Depression Rating Scale and/or the Tyrer Brief Anxiety Scale	The baseline using the Montgomery-Asberg Depression Rating Scale was 19.8 in the control group and 30 in the treatment group. The baseline using the HADS was 14.6 and 15.3 in the control and treatment group, respectively.

[24]	Double blind RCT	42	73, (44–85)	Female (32), male (10)	Individuals with cancer	Individuals with cancer with a wide variety of levels of physical and psychological symptoms	Baseline score using HADS was not stated. Only the median change in HADS was provided being 0 for the aromatherapy group, −1.5 for the massage group, −0.5 for the aromatherapy massage group, and 0.5 for the control.

[25]	RCT	288	52.1; 52.8 for the usual care group; and 51.5 for the usual care plus aromatherapy group	Female (250), male (38)	Individuals with cancer	Patients diagnosed with cancer, a prognosis of more than 3 months, with clinical anxiety or depression	The baseline score using the Center for Epidemiological Studies Depression Scale was 26.1 for the aromatherapy group and 26 for the group receiving usual care (control).

[26]	Nonblinded randomized crossover trial	16	46.1 (37.9–54.3)	Female (15), male (1)	Patients diagnosed with idiopathic environmental intolerance	Clinical examination by a physician and scoring above 26 for men and 30 for women in the Chemical Odor Sensitivity Scale	Depression subscale baseline score using POMS was around 2.8 in the control period.

[19]^*∗*^	Randomized observational pilot study with repeated measures	28	NA	Female (28)	Postpartum women	0–18-month postpartum women with scores of 10 or higher on either the Edinburgh Postnatal Depression Scale or the Generalized Anxiety Disorder Scale	The baseline score using the Edinburgh Postnatal Depression Scale for the control group was 15.9 and 16.1 for the intervention group.

[27]	Single blind RCT	39	52.5; 51.1 for the aromatherapy group; and 54 for the cognitive behavior therapy group	Female (31), male (8)	Individuals with cancer	Patients diagnosed for at least one month, who also had at least a predicted survival of 6 months and score 11 or more in the HADS for anxiety or depression	The baseline score in the depression-dejection subscale of POMS was 11.2 for the aromatherapy massage group and 13.4 for the control group.

[28]	RCT	90	53.70 for the control group (49.42–57.98), 52 (47.12–56.88) for the massage therapy group, and 53.35 (49.01–57.69)	Female (90)	Women who entered their menopausal period naturally	Woman, age between 45 and 60 years, with amenorrhea for at least 1 year	At baseline, according to the Menopause Rating Scale, the frequency of the severity of the depressive mood was reported as mild (14.9%), moderate (36.8%), severe (20.7%), and very severe (2.3%). No difference was found among the groups at baseline.

[29]	RCT	25	34–48, average age NA	Female (25)	Women with children	Women whose children were diagnosed with attention deficit hyperactivity disorder	Baseline using the Beck Depression Inventory was 8.6 in the control group and 10.8 in the treatment group.

^*∗*^In this study, both aromatherapy modalities were tested, inhalation aromatherapy and aromatherapy massage. Therefore, the study was included in both categories in the table. NA, not available; HADS, Hospital Anxiety and Depression Scale; POMS, Profile of Mood States.

**Table 3:** Description of the interventions and protocols used in the selected studies.

Reference	Intervention and protocol							
	Comparison group (*n*)	Treatment group(s) (*n*)	Type of essential oil used	Duration of the study	Administration method	Treatment frequency	Duration per session	Total number of sessions
*Inhalation aromatherapy*								

[18]	Control with sweet almond cold-pressed pure vegetable oil with no fragrance (NA)	(i) Carrier oil with fractionated low (NA) grade essential oil (ii) Pure essential oil (NA)	(i) Fractionated oils of unknown purity diluted 1 : 3 in carrier oil (ii) Mixture of lavender, bergamot, and cedarwood (2 : 1 : 1)	8 weeks	3 drops of oil applied to a bib worn during the administration of the treatment	Daily	15–20 min	56

[19]^*∗*^	Control, jojoba oil (14)	(i) 2% dilution of a mixture of essential oils (6)	(i) 0.25 rose otto essential oil and 0.75 lavender, 2% dilution of the essential oil mixture	4 weeks	8 drops of oil applied to a cotton pad. Subjects were instructed to smell the cotton pad for 15 min	Twice a week	15 min	8

[20]	Control, no intervention (6)	(i) Pure essential oil (7)	(i) Lavender (ii) Petitgrain (iii) Bergamot	1 day	5 drops of oil applied on a filter placed in a diffuser	Once	5 min	1

[21]	Control, water (20)	(i) Pure essential oil (20)	(i) Yuzu	2 days	10 *μ*L oil in a cotton pad used in a diffuser set in the subject's nostrils	Twice	10 min (sessions separated in intervals around 2.6 days)	2

[22]	Control group which did not receive any nonpharmacological method for pain relief of labor (160)	(i) Nonpharmacological methods for pain relief of labor including showering, being in upright position, aromatherapy, and soft music without words (160)	(i) 20% lavender essential oil	During labor	10 × 10 cm cloth impregnated with 1 mL 20% lavender essential oil which was attached to the mother's breast at the beginning of the active phase. The aromatherapy intervention was combined with other nonpharmacological interventions	Once	Duration of the active phase of labor	1

*Aromatherapy massage*								

[23]	Control, grape seed oil (16)	(i) Diluted essential oil (16)	(i) 9 essential oils (bergamot, lemon clary sage, lavender, roman chamomile, geranium, rose otto, sandalwood, and jasmine). A combination of essential oils chosen by the aromatherapist on each treatment session (16)	12 weeks	15 mL grape seed carrier oil with (4 drops) or without essential oils applied in a full body massage using gentle effleurage and petrissage	Once a fortnight	40 min	6

[24]	Control, no intervention (13)	(i) Aromatherapy massage (16) (ii) Massage with inert carrier oil (13)	(i) 1% lavender essential oil diluted in sweet almond oil	2 years	Back massage	Weekly	30 min	4

[25]	Usual supportive care	(i) Usual supportive care and aromatherapy massage	(i) 20 essential oils	10 weeks	Standardized massage agreed by the therapists	Weekly	1 h	4

[26]	Control, no intervention	(i) Aromatherapy massage	(i) 1% massage oil containing melissa, juniper, and rosemary essential oils mixed into jojoba oil (1 : 2 : 2 ratio)	8 weeks	Standardized massage on the back, shoulders, arms, hands, lower legs, and feet using 20–30 mL massage oil	Every two weeks	1 h	4

[19]^*∗*^	Control, essential oil blend unscented white lotion (14)	(i) 2% dilution of a mixture of essential oils (8)	(i) 0.25 rose otto essential oil and 0.75 lavender, 2% dilution of the essential oil mixture	4 weeks	Topic application of the oil or lotion on both hands with gentle strokes of homogeneous pressure and speed	Twice a week	15 min	8

[27]	Cognitive behavior therapy (19)	(i) Aromatherapy massage (20)	(i) 20 essential oils	2 years	Standardized massage combined with treatment as usual (routine support)	Weekly	1 h	Up to 8 sessions in 10 weeks

[28]	Control, no intervention (30)	(i) Aromatherapy massage (30) (ii) Massage (30)	(i) 3% oil mixture containing lavender, geranium, rose, and rosemary (4 : 2 : 1 : 1 ratio) in almond and evening primrose oil	4 weeks	Massage in the abdomen, tights, and arms using massage oil containing essential oils or odorless liquid petrolatum. Massage was applied with clockwise circular movements and light pressure	Twice a week	30 min	8

[29]	Control, no intervention (12)	(i) Aromatherapy massage (13)	(i) Jojoba oil containing 2% lavender and 2% geranium essential oils	4 weeks	Massage on the neck, shoulders, arms, back, and legs including effleurage, friction, petrissage, and vibration at a moderate pressure using 20 mL of massage oil	Twice per week	40 min	8

^*∗*^In this study, both aromatherapy modalities were tested, inhalation aromatherapy and aromatherapy massage. Therefore, the study was included in both categories in the table. NA, not available; min, minutes; h, hour.

**Table 4:** Description of the measurement tools, outcomes, and conclusions.

Reference	Outcome measures				
	Scale	Comparison group	Intervention group(s)	Outcome	Improvement of depressive symptoms
*Inhalation aromatherapy*					

[18]	(i) HADS	Control group	(i) Carrier oil with fractionated low grade essential oil (ii) Pure essential oil	Increased outcome measurement when compared to the baseline (18%–22% in HADS) using multivariate analysis. No statistically significant difference observed.	No

[19]^*∗*^	(i) EPDS	Control group	(i) 2% dilution of a mixture of essential oils	The mean difference in EPDS scores between the control group and the intervention group at end point was −3.981 but no statistically significant difference was observed. Combined analysis of inhalation aromatherapy and massage aromatherapy showed statistically significant difference with a mean difference of −4.8.	Yes

[20]	(i) POMS	No intervention	(i) Pure essential oil	Depression-dejection subscale scores before and after test were 2.7 and 1.2 in the comparison group and 1.6 and 0.6 in the intervention group, respectively. No statistically significant difference was observed.	No

[21]	(i) POMS in TMD	Control group	(i) Pure essential oil	Change in TMD score was 0.5 ± 2.2 in the control group and −1.28 ± 2.6 in the intervention group (statistically significant difference).	Yes

[22]	(i) EPDS	No intervention	(i) Nonpharmacological methods for pain relief of labor including showering, being in upright position, aromatherapy, and soft music without words	Depression grades (0–30) before and after delivery were 6.3 and 8.8 in the no intervention group and 6.1 and 7.8 in the intervention group, respectively. No statistically significant difference was observed.	No

*Aromatherapy massage*					

[23]	(i) MADRS	Control group	(i) Diluted essential oil	The scores in the MADRS at baseline and end point were 19.8 and 21.1 in the control group and 30 and 18.1 in the treatment group. Statistically significant difference was observed between the test and control group.	Yes

[24]	(i) HADS	No intervention	(i) Aromatherapy massage (ii) Massage with inert carrier oil	The median change in HADS at baseline and end point was 0.5 in the no intervention group, 0 in the aromatherapy massage group, and −1.5 in the massage group. No statistically significant difference among the groups.	No

[25]	(i) CES-D	Active control (usual supportive care)	(i) Usual supportive care and aromatherapy massage	The scores using CES-D at baseline and end point were 26.0 and 4.6 in the active control group and 25.9 and 6.2 in the intervention group, respectively. No statistically significant difference observed between the 2 groups.	No

[26]	(i) POMS	No intervention	(i) Aromatherapy massage	Statistically significant difference between the 2 groups when comparing the pre- and postsessions in all the POMS subscales including depression-dejection.	Yes

[19]^*∗*^	(i) EPDS	Control group	(i) m'technique (hand massage)	The mean difference between the baseline and end point using EPDS was −6.031.	Yes

[27]	(i) POMS-TMS	Active control (cognitive behavior therapy)	(i) Aromatherapy massage	The POMS-TMS decreased in both groups after intervention from 46.3 to 26.5 in the active control group and 44.5 to 29 in the intervention group.	Yes

[28]	(i) MRS	No intervention	(i) Aromatherapy massage (ii) Massage	The mean difference in psychological symptoms (including depressive mood) was −0.379 in the no intervention group (no statistically significant difference), −3.49 in the aromatherapy massage group (statistically significant difference), and −1.20 in the massage group (statistically significant difference).	Yes

[29]	(i) BDI	No intervention	(i) Aromatherapy massage	The BDI score before and after test was 8.6 and 8.5 in the control group and 10.8 and 6.5 in the intervention group (statistically significant difference).	Yes

^*∗*^In this study, both aromatherapy modalities were tested, inhalation aromatherapy and aromatherapy massage. Therefore, the study was included in both categories in the table. CES-D, Center of Epidemiological Studies Depression (self-reported depression); BDI, Beck Depression Inventory; DSM-IV, modified Diagnostic and Statistical Manual of Mental Disorder criteria; EPDS, Edinburgh Postnatal Depression Scale; HADS, Hospital Anxiety and Depression Scale; MADRS, Montgomery-Asberg Depression Rating Scale; MRS, Menopause Rating Scale; POMS, Profile of Mood States; TMD, Total Mood Disturbance; TMS, Total Mood Score (shortened version of the profile of mood states).

**Table 5:** Quality assessment of studies included according to the Jadad scale.

Reference	Quality assessment of methodology based on Jadad scale							
	Randomization	Appropriate method of randomization and description	Blinding				Description of dropouts/withdrawals	Jadad score (score out of 5)
			No blinding	Single blind	Double blind	Appropriate method of double blinding and description		
*Inhalation aromatherapy*								
[18]	Yes	Yes	No	No	Yes	Yes	No	4
[19]^*∗*^	Yes	No	Yes	No	No	NA	Yes	2
[20]	Yes	Yes	Yes	No	No	NA	Yes	3
[21]	Yes	No	No	Yes	No	No	No	1
[22]	Yes	Yes	No	No	Yes	No	Yes	4

*Aromatherapy massage*								
[23]	Yes	No	Yes	No	No	NA	No	1
[24]	Yes	Yes	No	No	Yes	Yes	Yes	5
[25]	Yes	Yes	No	No	Yes	No	Yes	3
[26]	Yes	Yes	Yes	No	No	NA	NA	2
[19]^*∗*^	Yes	No	Yes	No	No	NA	Yes	2
[27]	Yes	Yes	No	Yes	No	No	Yes	3
[28]	Yes	Yes	Yes	No	No	No	No	2
[29]	Yes	No	Yes	No	No	NA	No	1

^*∗*^In this study, both aromatherapy modalities were tested, inhalation aromatherapy and aromatherapy massage. Therefore, the study was included in both categories in the table. NA, not applicable.
